# Supplementary material for: Acceptability of an extended duration vaginal ring for HIV prevention and interest in a multi-purpose ring
Source: PLoS One. 2022 Feb 22;17(2):e0263664. doi: 10.1371/journal.pone.0263664 (PMC8863232; doi:10.1371/journal.pone.0263664)

MTN 038

In-depth interview topic guide

INSTRUCTIONS for the Interviewer: How to use the IDI Guide

1. The guide is divided by sections based on interview activity. The guide does NOT include very many transition sentences; however, the interviewer should use their discretion to insert transition language between questions or topics as needed.
2. Not ALL questions need to be asked. It is up to the interviewer's discretion if a question should be skipped if the participant has already provided a response to the question earlier in the interview. Please ensure that by the end of the interview, all the topics and key themes have been covered.
3. REMEMBER: Be CURIOUS! The more interested you are in the details of the participant's experiences, the richer the data we will get. We want to know the WHY behind her behaviors and her decisions regarding her ring use and study experience. If you think something is unclear, ask a follow-up question.
4. *Instructions/suggestions to interviewer are in italics and [brackets].*
5. There are two levels of questions:
    - Primary interview questions: appear in **bold** text. They address the topics that you as the interviewer should ask and discuss with participants. You are not required to read them verbatim, but they are written to ensure some consistency across IDIs.
    - Probing topics are indicated with a bullet. If you find that the participant does not provide much information in response to the primary question, these probing topics may be used to encourage further discussion. Depending on what has already been discussed, and the IDI context, you may or may not ask the rest of the probes.

- *Before starting the IDI, ensure the participant has provided verbal agreement to participate in the interview.*
Section 1: Introduction
[*Start Recorder and Read Introduction*]: **My name is __________________. Thank you for agreeing to complete an interview. I am looking forward to hearing about your experiences in this study. Please know there are no right or wrong answers and we welcome every opinion about the topics we will discuss, so feel free to share your thoughts, opinions, and views openly.**<br><br>**The main goal of this discussion is to better understand how the ring has worked out for you and how you have been using it. I want to remind you that sometimes we share quotes or summaries of information we learn during interviews, but it will never be able to be traced back to you. If we publish quotes from this interview, you will remain anonymous.**<br><br>**Before we start, can you confirm for the recorder that you agree to be interviewed today?** [*Wait for oral confirmation to begin*].<br><br>**1. First, I would like to hear a little about yourself and about your experience being part of the study. Overall, how has it been?**<br>• Experiences during study visits<br>• Effects of participation on daily life

Section 2: Experience with study product

2. **Now I want to talk more about the ring itself and your experiences using it during the study. What did you think about the ring when you saw it for the first time?**
    - Prior vaginal ring use or familiarity (e.g. Nuva Ring, Femring, etc.): impressions of comparison
    - Look/feel of the ring when she touched it
    - Thoughts about insertion process when she first heard about it
    - Thoughts about duration of use

3. **How did it feel once you actually started using the ring?**
    - Insertion process after she actually did it
    - Sensations after insertion, any change over time
    - Changes in how the vagina feels (wetter/drier)
    - Change in feelings about the ring after she started using it
    - Removal process

4. **What are, in your opinion, the possible benefits of using the ring?**
    - Advantages for others who may be at higher risk of HIV
    - Use during sex
    - Privacy/discreetness
    - Effects of the ring on the body
    - Changes in how the vagina feels (wetter/drier)
    - Hygiene and menstruation

5. **Looking back over the past 3 months, what did you personally like most about the ring you were given in this study?**
    - Convenience/ease of use
    - Dosing
    - Effects of the ring on her body
    - Physical characteristics of the ring
    - Partner attitudes
    - Hygiene and menstruation

6. **What are, in your opinion, the possible drawbacks of using the ring?**
    - Disadvantages for others who may be at higher risk of HIV
    - Use during sex
    - Privacy/discreetness
    - Effects of the ring on the body
    - Scents associated with ring use
    - Changes in how the vagina feels
    - Hygiene and menstruation

7. **And what did you personally like least about the ring?**
    - Challenge with use
    - Dosing
    - Effects of the ring on her body

- Physical characteristics of the ring
- Partner attitudes
- Hygiene and menstruation

8. What were your challenges with keeping the ring in continuously during the study?
- Explain circumstances/situations and whether typical for each of the following *[only probe on situations not already addressed in the previous two questions]*:
    - The ring coming out or being taken out
    - If removal/expulsion occurred: experience with reinsertion
    - Challenge during sex
    - Challenge during your menses or tampon use
    - Challenge during showering, bathing or going to the bathroom
    - Challenge doing sports or exercising

Section 3: Disclosure and effect on sex

9. Can you tell me about anyone who you talked to about the ring you were given in this study?
- Sex partner(s)
  [If YES]
    - How it went when they learned about the ring
    - Voluntary/involuntary disclosure
    - Initial reactions
    - Change over time

  [If NO]
    - What made you decide not to tell them about the ring?
    - Expected reactions of partner if they had been told/found out
    - If she would have disclosed ring use under different circumstances
- Other people:
    - Family
    - Friends
    - Other participants/study staff
    - Reasons for disclosure/non-disclosure
    - Anyone they are close to that they would not consider disclosing to

10. *[If not fully explored in #4 and #6]* **How, if at all, would you say that using this ring has influenced your sexual activity in the past few months?**
- Her awareness of the ring during sex and effect on her pleasure
- Changes in sexual practices, positions, partners, or frequency of sex due to ring use
- Effects of ring use on sexual partner(s) and their pleasure

Section 4: Product preferences

11. How does the ring compare with other products that can prevent HIV?
- What kinds of products she has used (male or female condoms, oral PrEP/ Truvada, etc.)
- What other products she knows about but has not used (male or female condoms, Truvada, etc.)
- How the ring is similar or different from these other products
- Supposing that the ring was equally effective, which she would rather use and why?

- How she would compare the ring to hypothetical products such as a 3-month injection or a 6-12 month implant?

12. **How do you feel about using the same ring for 3 months continuously versus using a new ring each month?**
    - Benefits and drawbacks to a longer duration
    - Benefits and drawbacks for a shorter duration
    - Concerns with each option
    - Ideal duration preference and why

13. **There are a variety of different ring designs that are being developed. Please look at the ring visual aids. The ring you used is labeled with a "1". The other rings are made from different materials and have different sizes. Let's discuss each of the other rings one by one, thinking aloud as you explain what you think about each one** *[go over probes below for Ring #2, Ring #3 etc.]*.
    - Flexibility
    - Texture
    - Circumference and width
    - Look/ color
    - Attributes that she would have preferred
    - Attributes that she finds undesirable

14. **Now that you have seen some alternative ring designs, what would you change about the ring you used during this study to improve it?**
    - Aside from attributes of alternative rings presented, other aspects she would change
    - How small is too small? How thin is too thin? Etc.

15. **Researchers are also working to develop a ring like this that would prevent HIV and pregnancy at the same time, it's called a multipurpose prevention technology, or "MPT" ring. What would you think about a MPT ring?**
    - Main concerns
    - Benefit of a dual purpose vaginal ring
    - If it is not right for them: Who (which types of women) would it appeal most to and why
    - Other MPT products she would prefer to use compared to a ring (condoms, hypothetical: implant, injection, oral pill)

Section 5: Closing

16. **Thank you for your insight and feedback on the ring so far. Before we finish, do you have any questions for me or if you have any additional comments about your experience in this study?**

[Thank participant for their time and responses.]

Appendix 1: Ring Comparison Tool

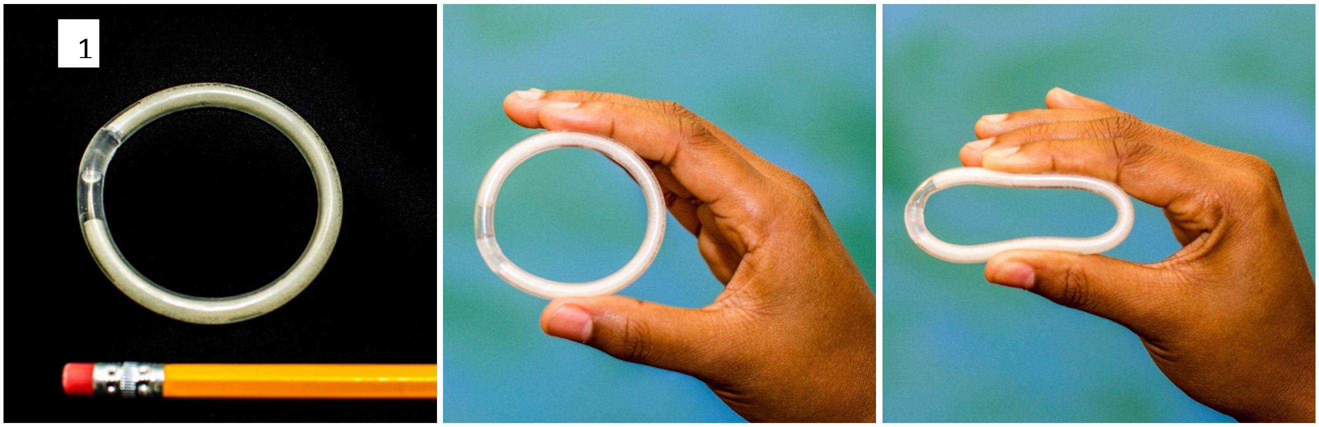

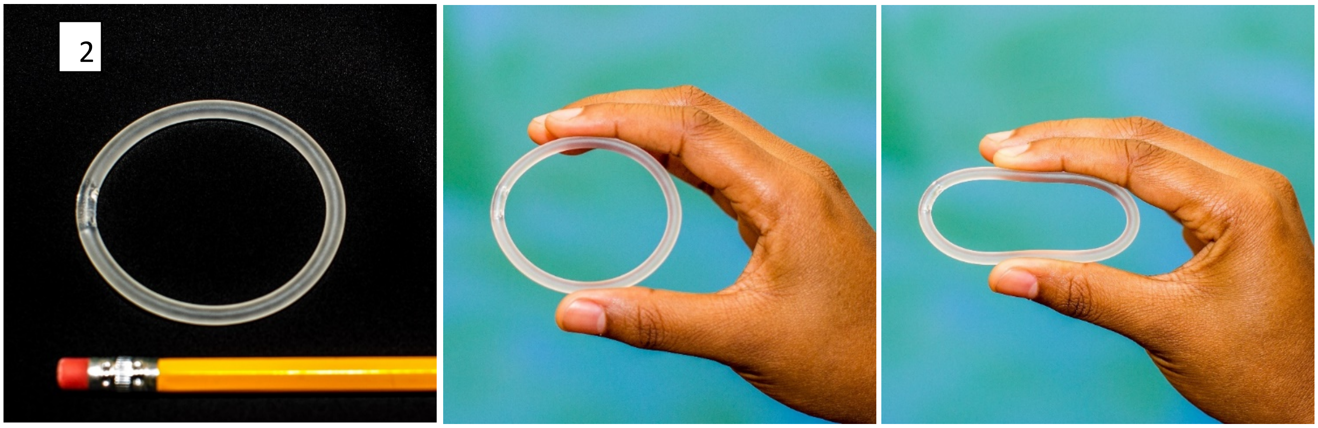

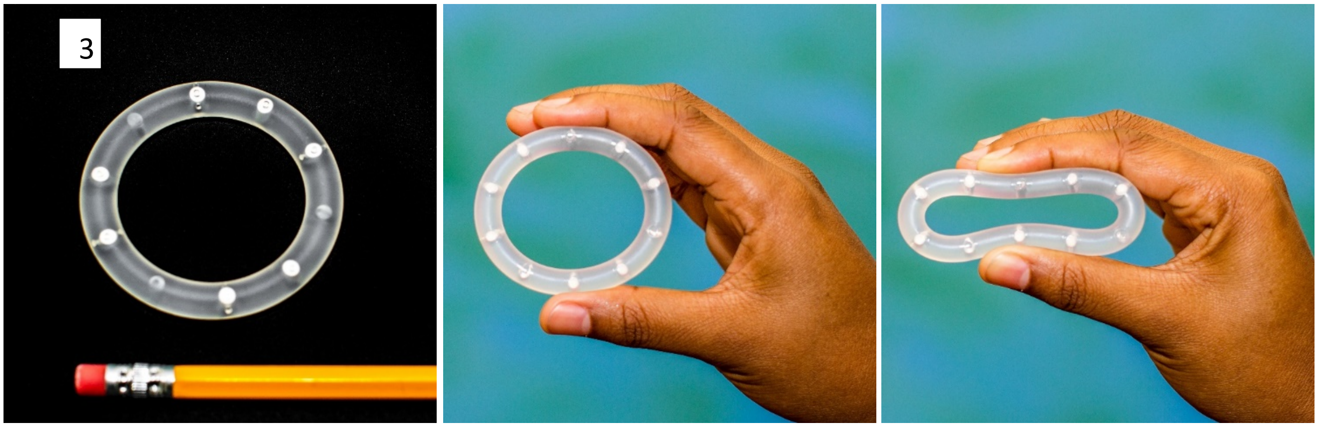

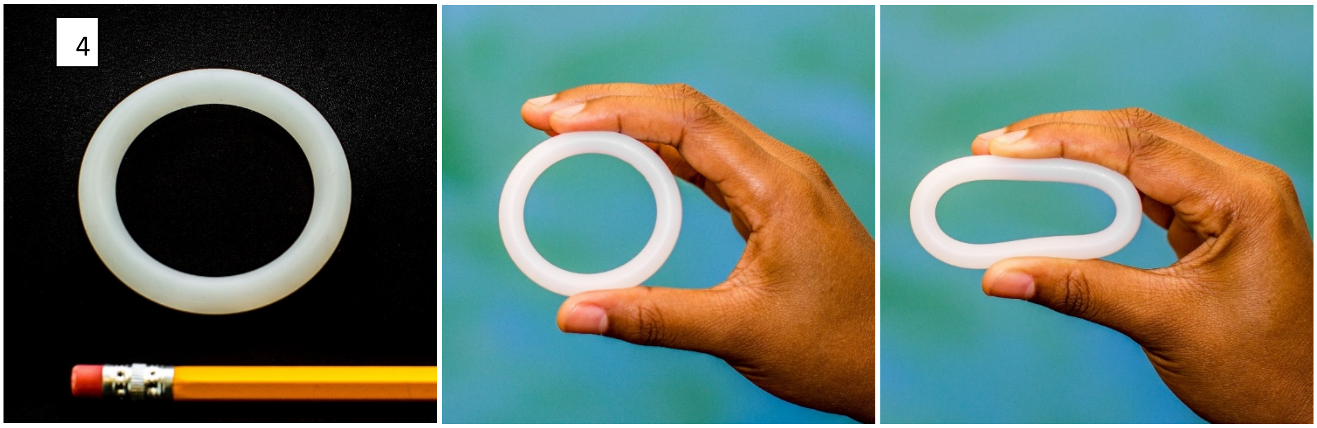

Supplement: S1 File — (PDF) [file pone.0263664.s001.pdf]
